# The impact of rumen microbial composition on apparent digestibility, rumen fermentation and metabolism in Sanhe cows and Holstein cows of different parities under identical dietary conditions

**DOI:** 10.3389/fvets.2024.1463209

**Published:** 2025-02-17

**Authors:** Zixin Liu, Aoyu Jiang, Dianyu Ma, Dexin Liu, Xiaoyu Han, Man Zhao, Chuanshe Zhou, Zhiliang Tan

**Affiliations:** ^1^Key Laboratory for Agro-Ecological Processes in Subtropical Region, Institute of Subtropical Agriculture, Chinese Academy of Sciences, Changsha, China; ^2^University of the Chinese Academy of Sciences, Beijing, China; ^3^Hulun Buir State Farm Xieertala Farm and Ranch Co., Ltd., Hulunbuir, China

**Keywords:** rumen microbiome, cow breeds, prity, dairy cattle, lactation association

## Abstract

Previous studies have discussed the association between serum metabolism and lactation performance among Sanhe and Holstein cows of different parities and found that the metabolic profiles of these two breeds vary differently with parity. Since the rumen is the central organ for nutrient absorption and production transformation in dairy cows, it remains unknown whether the differences observed under the same dietary conditions are related to the structure of the rumen microbiome. This study measured the apparent digestibility and rumen fermentation parameters of Sanhe cows (S1/S2/S3/S4) and Holstein cows (H1/H2/H3/H4) across four parities and generated a comprehensive rumen microbiome dataset using high-throughput sequencing technology. Significant differences in dry matter digestibility (*p* = 0.001) and ammonia nitrogen (*p* = 0.024) were observed among the S groups, with higher trends of various VFA contents in S1 (0.05 < *p* < 0.1). The H group showed significant differences in crude protein digestibility (*p* = 0.001), higher isovaleric acid content in H1 (*p* = 0.002), and the lowest acetate to propionate ratio (*p* = 0.002) in H3. Metagenomic sequencing results indicated consistency between rumen microbiome patterns and metabolic changes, with S1 distinctly different from S2/S3/S4, and H1 and H2 different from H3 and H4. The species composition of the rumen microbiome was similar between Sanhe and Holstein cows, but differences in abundance were noted. *Rhizophagus <glomeromycetes>*, *Neocallimastix*, and *Piromyces* were more abundant in S1, H1, and H2, and pathways such as autophagy-animal, plant-pathogen interaction, and endocytosis were significantly enriched in these parities. Multiparous Sanhe cows had higher abundances of ATP-binding cassette transporters pathways. Additionally, CAZymes such as GH84 and GH37 were significantly associated with differential physiological indicators and milk traits. In conclusion, this study reveals the complex relationship between rumen microbiota and metabolic characteristics in Sanhe and Holstein cows of different parities, indicating that changes in the structure of the rumen microbiome may be key factors affecting lactation performance and metabolic differences in dairy cows.

## 1 Introduction

In recent years, with the increasing demand for high-quality dairy and beef products, cattle breeding and management have garnered growing attention worldwide. Among the numerous cattle breeds, Holstein cows are one of the most widely distributed and extensively studied dairy breeds globally (1). However, there is also a rich diversity of genetic resources in cattle breeding and production, including indigenous breeds such as the Sanhe cattle in China (2, 3). Through selective breeding, Sanhe cattle have been developed for both dairy and meat production. Nevertheless, compared to widely studied breeds like Holstein, there remains a significant gap in understanding the production performance and physiological characteristics of Sanhe cattle.

Lactation is the most critical production activity in dairy cows. Due to differences in basal metabolic levels and energy requirements among cows of different parities (4), the impact of parity on lactation performance cannot be overlooked under normal feeding and management conditions. In our previous research, we systematically explored the physiological and metabolic characteristics of Sanhe and Holstein cows across parities 1 to 4 (5). We found significant differences in the metabolic patterns between primiparous and multiparous Sanhe cows, indicating substantial changes in nutrient utilization and lactation-related metabolic pathways during continuous lactation periods. In Holstein cows, the metabolic profiles exhibited different trends, with the first and second parities being more similar to each other and distinct from the third and fourth parities (6).

For dairy cows, the composition and activity of the rumen microbiome are crucial factors determining feed efficiency, nutrient utilization, and overall productivity (7). However, most existing studies focus on the development of the rumen microbial community in weaned calves (8) and the differences in rumen microbiome composition between high-and low-producing dairy cows (9, 10). There is limited understanding of the successional patterns of the rumen microbiome in lactating cows of different parities. Xue et al. (11) found significant differences in the relative abundance of *Fibrobacteres* and *SR1* in the rumens of mid-lactation cows from parities 2 to 7, while the main functional metabolic bacteria showed no significant changes. The lactation traits of dairy cows have a complex relationship with the core rumen microbiota (12–14). Therefore, the rumen microbiome is a critical entry point for studying the mechanisms regulating milk quality in dairy cows.

We hypothesize that under identical dietary conditions, the differences in serum metabolism and lactation performance between Sanhe and Holstein cows of different parities are partially attributable to specific changes in their rumen microbial community structure. This study focuses on investigating the impact of rumen microbial composition on rumen fermentation and metabolic activities in Sanhe and Holstein cows across parities 1 to 4 under the same dietary conditions. The aim is to further elucidate the effects of rumen microecology on metabolic adaptability and production performance in specific breeds of dairy cows. Combined with previous research findings, this study can provide a comprehensive framework to clarify the multifaceted interactions among host physiology, rumen microbiota, and metabolic responses. This will enhance the understanding of the Sanhe breed and inform breeding and management strategies in the context of sustainable livestock production.

## 2 Materials and methods

### 2.1 Animal management and experimental design

All procedures involving animals in this experiment were approved by the Animal Experiment Ethics Committee of the Institute of Subtropical Agriculture, Chinese Academy of Sciences. Sanhe and Holstein cows in lactation periods from parity 1 to 4 were selected from the same farm, with similar feeding management conditions and identical dietary formulations. The four groups of Sanhe cows were designated as S1 (*N* = 10), S2 (*N* = 9), S3 (*N* = 10), and S4 (*N* = 10), while the four groups of Holstein cows were designated as H1 (*N* = 10), H2 (*N* = 7), H3 (*N* = 9), and H4 (*N* = 9). Detailed information on feeding management procedures and animal selection can be found in our previously published works, including studies on Sanhe cows (5) and Holstein cows (6).

### 2.2 Collection and analysis of fecal samples

During the sampling period, rectal fecal samples were collected for five consecutive days, twice daily, 2 h before feeding and 2 h after feeding. The samples were mixed with 10% sulfuric acid and stored at −20°C until analysis for apparent digestibility. Fecal samples were dried at 65°C and ground to pass through a 1-mm sieve. The measurements of dry matter (DM; method 930.15), crude protein (CP; method 2001.11), neutral detergent fiber (NDF; method 2002.04), acid detergent fiber (ADF; method 973.18), and ether extract (EE; method 920.39) were conducted following the methods of the Association of Official Analytical Chemists (55, 56). Gross energy (GE) was determined using a calorimeter (5E-C5508, Kaiyuan Instruments, China). Apparent total-tract digestibility was calculated using the acid-insoluble ash method (15).

### 2.3 Collection and analysis of rumen samples

Using an oral intubation method, a tube was inserted approximately 120–150 cm deep into the esophagus, and contents from the rumen were aspirated using a syringe at the oral end. To avoid saliva contamination, the initial 100 mL of aspirated content was discarded, and the subsequent 100 to 150 mL of rumen fluid was collected. Immediately on-site, the pH value was measured using a portable pH meter (BPHPOCKET-E, BELL Analytical Instruments (Dalian) Co., Ltd., China). The fluid was then filtered through two layers of sterile gauze into sterile centrifuge tubes and flash-frozen using liquid nitrogen. As described previously (16), after processing the rumen fluid, volatile fatty acid (VFA) concentrations were analyzed using Gas Chromatography (7890A, Agilent, United States). Ammonia nitrogen concentration was determined using a UV-2300 Spectrophotometer (Shimadzu, Kyoto, Japan) by recording absorbance at 700 nm.

### 2.4 Extraction of rumen fluid DNA and metagenomic sequencing

The extraction of rumen fluid DNA was performed by Guangdong Magigene Biotechnology Co., Ltd. (Guangzhou, China) using a commercial kit following the manufacturer’s instructions. The integrity of the DNA was checked using 1% agarose gel electrophoresis. DNA concentration and purity were assessed simultaneously using the Qubit 2.0 (Thermo Fisher Scientific, Waltham, United States) and Nanodrop One (Thermo Fisher Scientific, Waltham, United States). Sequencing libraries were prepared using the NEB Next^®^ Ultra^™^ DNA Library Prep Kit for Illumina^®^ (New England Biolabs, Ipswich, MA, United States) following the manufacturer’s recommendations, with index codes added. Library quality was evaluated using the Qubit 3.0 Fluorometer (Life Technologies, Grand Island, NY) and the Agilent 4200 (Agilent, Santa Clara, CA) system. Finally, the libraries were sequenced on the Illumina Hiseq X-ten platform, generating 150 bp paired-end reads.

### 2.5 Bioinformatic processing of rumen metagenomic data

The raw data obtained from sequencing were processed using Trimmomatic (v.0.36, http://www.usadellab.org/cms/index.php?page=trimmomatic) to obtain clean data for subsequent analysis. Clean data were assembled using MEGAHIT (Version v1.0.6, https://github.com/voutcn/megahit). Mixed assembled scaffolds were broken from N connection to obtain scaftigs. Scaftigs ≥500 bp were screened and used for ORF prediction with MetaGeneMark (Version 3.38, http://exon.gatech.edu/GeneMark/metagenome/Prediction), with default parameters filtering out predicted results shorter than 90 nt. Redundancies were removed using CD-HIT (Version 4.7, http://www.bioinformatics.org/cd-hit/), creating a unique initial gene catalogue. Clustering was performed with 95% identity and 90% coverage, selecting the longest representative sequence. Clean data from each sample were mapped to the initial gene catalogue using BBMAP,^1^ obtaining gene mapping reads for each sample. Gene abundance information for each sample was calculated based on the number of mapped reads and the gene length. DIAMOND software (Version 0.8.35, https://github.com/bbuchfink/diamond/) was used to align unigenes against bacterial, fungal, archaeal, and viral sequences extracted from the NCBI NR (non-redundant protein sequence database). Results with an *e*-value of 1 × 10^−10^ were selected for LCA algorithm annotation. Based on the LCA annotation results and gene average depth or gene abundance tables, gene average depth and abundance information tables for each taxonomic level (kingdom, phylum, class, order, family, genus, species) were obtained for each sample. Abundance clustering heatmaps and PCA were based on abundance tables at each taxonomic level. Group differences were tested using ANOSIM analysis. LEfSe analysis (default LDA score of 2) was used to identify different species between groups. Visualization was performed using the R package ggplot2 (version 4.3.1). Clustering heatmaps were generated using the R package Pheatmap. Differential KEGG pathways were visualized using OmicStudio tools.^2^ CAZy^3^ annotation results were generated by dbCAN^4^ to obtain annotation information of carbohydrate-active enzymes. CAZy classification circular heatmaps and bar charts were created using the online analysis tool ChiPlot.^5^ Spearman correlation analysis was used to analyze the differential GH enzymes and apparent differential indices, with visualization performed using the linkET and ggplot2 packages.

### 2.6 Statistical analysis

Data for apparent digestibility and rumen fermentation parameters were initially organized using Excel 2019 (Microsoft Corporation, United States) and then subjected to statistical analysis using Statistical Package for the Social Sciences 22.0 software (SPSS, Inc., United States). The normality of each variable was assessed with the Shapiro–Wilk test. If the data met the normal distribution criteria, comparisons were made using analysis of variance (ANOVA), and multiple comparisons between categorical variables were adjusted using Bonferroni correction. A *p-*value <0.05 was defined as statistically significant, 0.05 ≤ *p* < 0.10 was defined as a trend, and *p* ≥ 0.10 was defined as no difference.

## 3 Results

### 3.1 Nutrients digestibility

The apparent digestibility results for Sanhe cows from parity 1 to 4 are shown in Table 1. The digestibility of DM differed significantly among the four parities, with S4 being lower (*p* = 0.038). However, the digestibility of other nutritional indices, including CP, NDF, ADF, EE, and GE, showed no significant differences between S1 and S4 (*p* > 0.05). The digestibility results for Holstein cows from parity 1 to 4 are presented in Table 2. Except for the CP digestibility, which was significantly higher in H4 compared to H1, H2, and H3, the digestibility of DM, NDF, ADF, EE, and GE showed no significant differences among H1 to H4 (*p* > 0.05).

**Table 1:** Apparent total-tract apparent digestibility of nutrients in Sanhe cows with 1–4 parities.

Item^1^	Group^2^				SEM^3^	*p*-value
	S1	S2	S3	S4		
DM	86.42^a^	85.59^ab^	85.96^a^	84.03^b^	0.325	0.038
CP	63.80	59.72	62.63	59.88	0.832	0.220
NDF	56.98	52.86	58.96	50.91	1.201	0.059
ADF	47.47	44.49	48.52	40.14	1.642	0.260
EE	85.37	82.49	83.34	80.79	0.707	0.134
GE	58.72	52.41	56.74	52.10	1.061	0.061

1 DM, dry matter; CP, crude protein; NDF, neutral detergent fiber; ADF, acid detergent fiber; EE, ethanol extract; GE, gross energy.

2 S1, S2, S3, and S4 represented first-, second-, third-, and fourth-parity Sanhe dairy cattle, respectively.

3 SEM was standard error of means.

^a,b^Means within a row with different superscripts differ significantly (*p* < 0.05).

**Table 2:** Apparent total-tract apparent digestibility of nutrients in Holstein cows with 1–4 parities.

Item^1^	Group^2^				SEM^3^	*p*-value
	H1	H2	H3	H4		
DM	84.01	83.31	83.07	84.96	0.385	0.279
CP	62.72^b^	62.36^b^	65.40^b^	69.58^a^	0.782	0.001
NDF	57.12	55.76	48.19	55.55	1.498	0.134
ADF	59.55	57.04	50.81	56.55	1.448	0.169
EE	85.65	84.37	83.14	83.79	0.613	0.507
GE	58.22	56.45	53.98	59.93	1.006	0.172

1 DM, dry matter; CP, crude protein; NDF, neutral detergent fiber; ADF, acid detergent fiber; EE, ethanol extract; GE, gross energy.

2 S1, S2, S3, and S4 represented first-, second-, third-, and fourth-parity Holstein dairy cattle, respectively.

3 SEM was standard error of means.

^a,b^Means within a row with different superscripts differ significantly (*p* < 0.05).

### 3.2 Rumen fermentation parameters

The rumen fermentation parameters for Sanhe cows from parity 1 to 4 are shown in Table 3. There was a trend for lower rumen pH in S1 (*p* = 0.068). Conversely, ammonia N was significantly higher in S1 and lowest in S2 (*p* = 0.024). Additionally, the mean values for total VFA, acetate, butyrate, isobutyrate, valerate, and isovalerate were higher in S1 (0.05 < *p* < 0.1). There were no significant differences in MCP and acetate/propionate ratios among S1 to S4 (*p* > 0.05). The rumen fermentation parameters for Holstein cows from parity 1 to 4 are presented in Table 4. The acetate/propionate ratio was significantly lower in H3 (*p* = 0.002), whereas isovalerate was significantly higher in H1 (*p* = 0.002). Other rumen fermentation parameters showed no significant differences among H1 to H4 (*p* > 0.05).

**Table 3:** Rumen fermentation variables in Sanhe cows with 1–4 parities.

Item^1^	Group^2^				SEM^3^	*p*-value
	S1	S2	S3	S4		
Rumen pH	6.52	6.77	6.73	6.56	0.040	0.068
Ammonia N (mg/dL)	8.59^a^	5.20^b^	6.67^ab^	7.75^a^	0.423	0.024
MCP (mg/mL)	1.42	1.45	1.37	1.21	0.064	0.547
Total VFA (mmol/L)	92.19	63.74	81.69	88.61	3.971	0.053
Acetate (mmol/L)	57.03	40.41	49.82	53.91	2.300	0.058
Propionate (mmol/L)	19.52	12.89	18.16	20.11	1.086	0.079
Butyrate (mmol/L)	11.91	7.79	10.26	11.08	0.565	0.056
Isobutyrate (mmol/L)	0.87	0.64	0.81	0.80	0.031	0.059
Valerate (mmol/L)	1.48	1.03	1.39	1.43	0.068	0.077
Isovalerate (mmol/L)	1.38	0.98	1.26	1.29	0.055	0.070
Acetate/Propionate	2.99	3.18	2.84	2.89	0.073	0.386

1 MCP, microbial crude protein; VFA, volatile fatty acid.

2 S1, S2, S3, and S4 represented first-, second-, third-, and fourth-parity Sanhe dairy cattle, respectively.

3 SEM was standard error of means.

^a,b^Means within a row with different superscripts differ significantly (*p* < 0.05).

**Table 4:** Rumen fermentation variables in Holstein cows with 1–4 parities.

Item^1^	Group^2^				SEM^3^	*p*-value
	H1	H2	H3	H4		
Rumen pH	6.71	6.61	6.69	6.83	0.050	0.521
Ammonia N (mg/dL)	9.23	6.99	7.86	6.33	0.489	0.176
MCP (mg/mL)	1.44	1.38	1.28	1.43	0.073	0.864
Total VFA (mmol/L)	96.53	78.20	83.17	74.23	4.590	0.336
Acetate (mmol/L)	60.74	49.22	49.71	46.53	2.744	0.253
Propionate (mmol/L)	20.37	16.61	21.19	16.45	1.221	0.396
Butyrate (mmol/L)	11.76	9.50	9.09	8.69	0.627	0.289
Isobutyrate (mmol/L)	0.88	0.67	0.80	0.61	0.056	0.315
Valerate (mmol/L)	1.40	1.11	1.41	1.08	0.070	0.175
Isovalerate (mmol/L)	1.38^a^	1.08^b^	0.96^bc^	0.88b^c^	0.054	0.002
Acetate/Propionate	3.05^a^	3.06^a^	2.43^b^	2.84^a^	0.074	0.002

1 MCP, microbial crude protein; VFA, volatile fatty acid.

2 S1, S2, S3, and S4 represented first-, second-, third-, and fourth-parity Holstein dairy cattle, respectively.

3 SEM was standard error of means.

^a,b^Means within a row with different superscripts differ significantly (*p* < 0.05).

### 3.3 Rumen microbial composition

**Figure 1:**
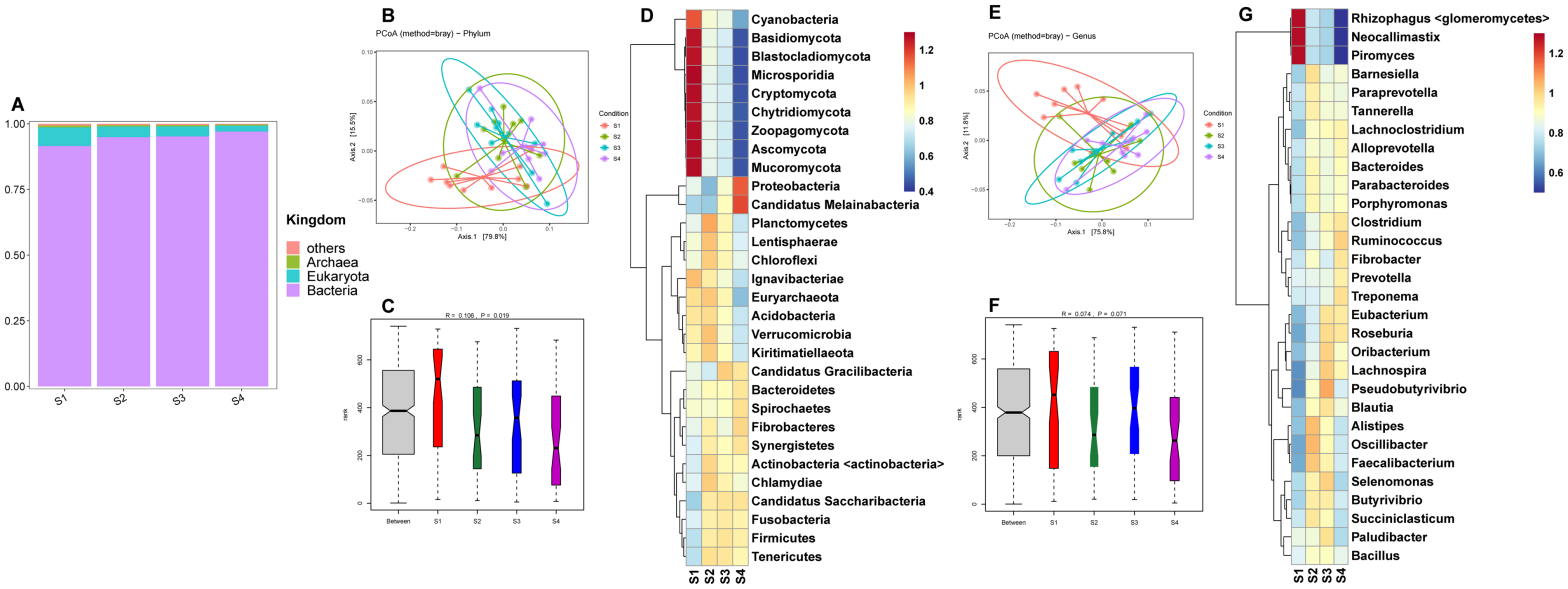
**(A)** Relative abundance distribution of microorganisms at the kingdom level in the rumen of S1–S4. **(B)** PCoA (principal coordinate analysis) clustering analysis of rumen microorganisms at the phylum level in S1–S4 based on the Bray method. **(C)** ANOSIM analysis of rumen microorganisms at the phylum level in S1–S4. **(D)** Heatmap of hierarchical clustering analysis of rumen microorganisms at the phylum level in S1–S4. **(E)** PCoA analysis of rumen microorganisms at the genus level in S1–S4 based on the Bray method. **(F)** ANOSIM analysis of rumen microorganisms at the genus level in S1–S4. **(G)** Heatmap of hierarchical clustering analysis of rumen microorganisms at the genus level in S1–S4.

A total of 2,712,982,832 clean reads were obtained from the 39 rumen samples of Sanhe cows (Supplementary Table S1). Microbial identification at the kingdom level revealed four types: bacteria, eukaryota, archaea, and others (Figure 1A). Among the S1–S4 parities, bacteria had the highest abundance, followed by eukaryota. The abundance of bacteria in S1 was slightly lower than in S3–S4, while eukaryota showed the opposite trend. The PCoA results for the 185 identified phylum-level species are shown in Figure 1B, indicating that the four groups were relatively clustered. However, ANOSIM analysis results demonstrated that inter-group differences were significantly greater than intra-group differences (Figure 1C), with S1 having the highest richness. Hierarchical clustering analysis (HCA) of the top 30 phylum-level microbes revealed that *Cyanobacteria*, *Basidiomycota*, *Blastocladiomycota*, *Microsporidia*, *Cryptomycota*, *Chytridiomycota*, *Zoopagomycota*, *Ascomycota*, and *Mucoromycota* had higher abundances in S1, while *Proteobacteria* and *Candidatus Melainabacteria* were more abundant in S4 (Figure 1D). Further analysis of the 3,528 identified genus-level microbes showed similar PCoA results to the phylum level, with less distinct separation (Figure 1E). ANOSIM analysis indicated a trend towards inter-group differences (Figure 1F). The HCA results for the top 30 genus-level species indicated that *Rhizophagus <glomeromycetes>*, *Neocallimastix*, and *Piromyces* were more abundant in S1 (Figure 1G).

**Figure 2:**
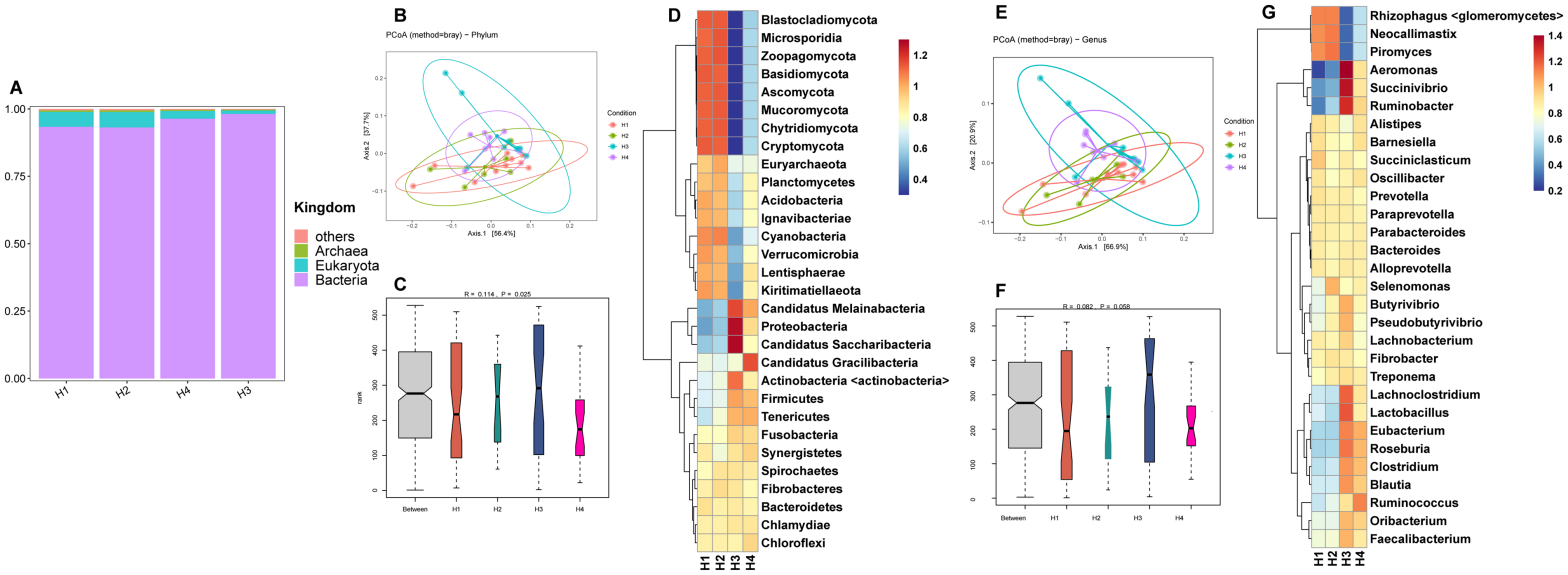
**(A)** Relative abundance distribution of microorganisms at the kingdom level in the rumen of H1–H4. **(B)** PCoA (principal coordinate analysis) clustering analysis of rumen microorganisms at the phylum level in H1–H4 based on the Bray method. **(C)** ANOSIM analysis of rumen microorganisms at the phylum level in H1–H4. **(D)** Heatmap of hierarchical clustering analysis of rumen microorganisms at the phylum level in H1–H4. **(E)** PCoA analysis of rumen microorganisms at the genus level in H1–H4 based on the Bray method. **(F)** ANOSIM analysis of rumen microorganisms at the genus level in H1–H4. **(G)** Heatmap of hierarchical clustering analysis of rumen microorganisms at the genus level in H1–H4.

The metagenomic sequencing data for rumen fluid from Holstein cows (H1–H4) are provided in Supplementary Table S2. The kingdom-level microbial composition structure was similar to that of Sanhe cows, with bacteria abundance being slightly lower in H1 and H2 compared to H3 and H4, and eukaryota showing the opposite trend (Figure 2A). Analysis of selected phylum-and genus-level microbes in H1–H4 revealed that PCoA showed no distinct separation between groups (Figures 2B,E). ANOSIM analysis indicated significant differences at the phylum level between groups (Figure 2C) and a trend towards differences at the genus level (Figure 2F). The HCA heatmaps of phylum-and genus-level microbes showed that the microbial abundance patterns of H1 and H2 were more similar. Specifically, phylum-level microbes such as *Blastocladiomycota*, *Microsporidia*, *Zoopagomycota*, *Basidiomycota*, *Ascomycota*, *Mucoromycota*, *Chytridiomycota*, and *Cryptomycota* had higher abundances in H1 and H2 (Figure 2D). Similarly, genus-level microbes such as *Rhizophagus <glomeromycetes>*, *Neocallimastix*, and *Piromyces* also had higher abundances in H1 and H2 (Figure 2G).

### 3.4 Differential rumen microbes and KEGG functional pathways

**Figure 3:**
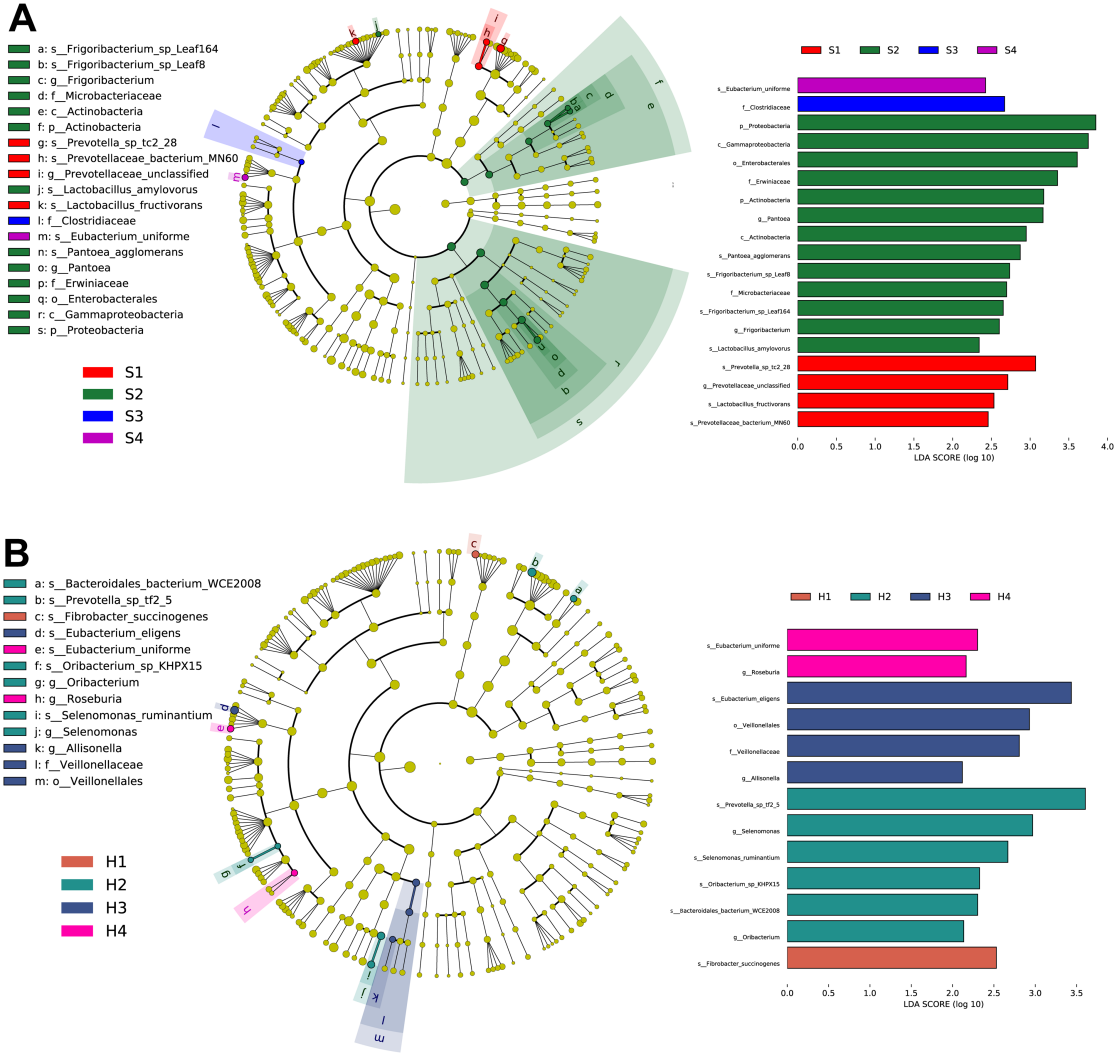
**(A)** Taxonomic tree and biomarker microorganisms of rumen microbiota in S1–S4 based on linear discriminant analysis effect size (LEFSe). **(B)** Taxonomic tree and biomarker microorganisms of rumen microbiota in H1–H4 based on LEFSe. Nodes of different colors represent representative microorganisms in the rumen of cows at different parities, with the specific names of the microorganisms labeled beside the nodes. The selection criteria for biomarkers are linear discriminant analysis (LDA) score >2.0, *p* < 0.05.

To further identify microbial composition differences and select representative marker microbes between different parities, we conducted LEfSe analysis on 1–4 parity Sanhe and Holstein cows. According to the LDA and evolutionary analysis results (Figure 3A), 19 marker microbes were identified in S1–S4 (*p* < 0.05). Notably, three of the four microbes enriched in S1 were related to *Prevotella* (*s_Prevotella_sp_tc2_28*, *g_Prevotellaceae_unclassified*, *s_Prevotellaceae_bacterium_MN60*), and the other was *s_Lactobacillus_fructivorans*. S2 had the highest number of marker microbes, including *p_Proteobacteria*, *c_Gammaproteobacteria*, *o_Enterobacterales*, *f_Erwiniaceae*, *p_Actinobacteria*, *g_Pantoea*, *c_Actinobacteria*, *s_Pantoea_agglomerans*, *s_Frigoribacterium_sp_Leaf8*, *f_Microbacteriaceae*, *s_Frigoribacterium_sp_Leaf164*, *g_Frigoribacterium*, and *s_Lactobacillus_amylovorus*, totaling 13. S3 and S4 had one significantly enriched microbe each, *s_Eubacterium_uniforme* and *f_Clostridiaceae*, respectively.

Similarly, in the LEfSe results for H1–H4 (Figure 3B), *s_Fibrobacter_succinogenes* was significantly enriched in H1. In H2, *s_Prevotella_sp_tf2_5*, *g_Selenomonas*, *s_Selenomonas_ruminantium*, *s_Oribacterium_sp_KHPX15*, *acteroidales_bacterium_WCE2008*, and *g_Oribacterium* were significantly enriched. H3 had significant enrichments in *s_Eubacterium_eligens*, *o_Veillonellales*, *f_Veillonellaceae*, and *g_Allisonella*. H4 had significant enrichments in *s_Eubacterium_uniforme* and *g_Roseburia*.

**Figure 4:**
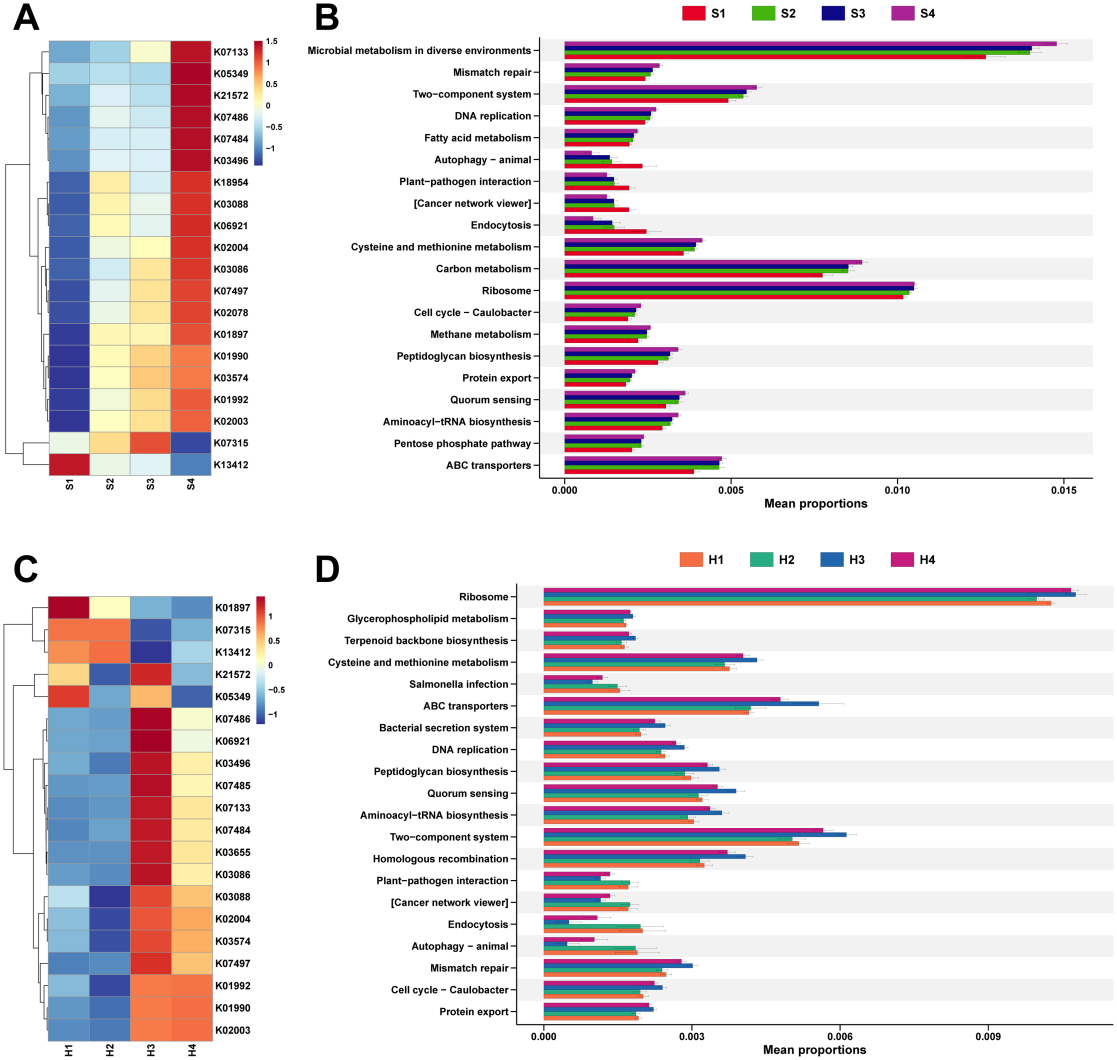
**(A)** Analysis of the top 20 differential KEGG Orthology (KO) identified in the rumen microbiota of S1–S4. **(B)** Analysis of the top 20 differential KEGG functional pathways identified in the rumen microbiota of S1–S4. **(C)** Analysis of the top 20 differential KO identified in the rumen microbiota of H1–H4. **(D)** Analysis of the top 20 differential KEGG functional pathways identified in the rumen microbiota of H1–H4. The stamp plots were created using the rank-sum test (Kruskal test), selecting the top 20 pathways by abundance with *p* < 0.05.

Continuing with the analysis of functional pathways enriched by these rumen microbiota. For the rumen fluid of multiparous Sanhe cows, a heatmap was generated for the top 20 identified KEGG orthologies (KO) number, revealing that, apart from K13412 showing higher expression in S1, the rest exhibited higher abundance in S2–S3 (Figure 4A).

It was also found that K01990, K01992, and K02003 are all related to ABC Transport, while K13412, which has a higher abundance in S1, is associated with calcium-dependent protein kinase (Supplementary Table S3). Stamp analysis was conducted on 425 level 3 pathways mapped to the KEGG database, selecting the top 20 pathways with significant differences in abundance (Figure 4B). Except for autophagy–animal, plant–pathogen interaction, cancer network viewer, and endocytosis showing higher expression in S1, other pathways with differential expression were more abundant in multiparous Sanhe cows.

Concurrently, analysis of microbial functional pathways in Holstein H1–H4 revealed that, apart from K21572 and K05349 showing higher abundance in H1 and H3, the remaining KO pathways exhibited more similarity in abundance between H1 and H2, whereas H3 and H4 showed similar patterns (Figure 4C). The KO pathways with higher abundance in H1 and H2 are mainly related to cell signaling and metabolic regulation, and their activity is influenced by phosphorylation status (Supplementary Table S4). Comparative analysis with the KEGG database identified 4 pathways significantly more abundant in S2 and also higher in abundance in H1 and H2, with salmonella infection additionally showing higher abundance in H1 and H2 (Figure 4D).

### 3.5 The composition of CAZymes in the rumen microbiota

**Figure 5:**
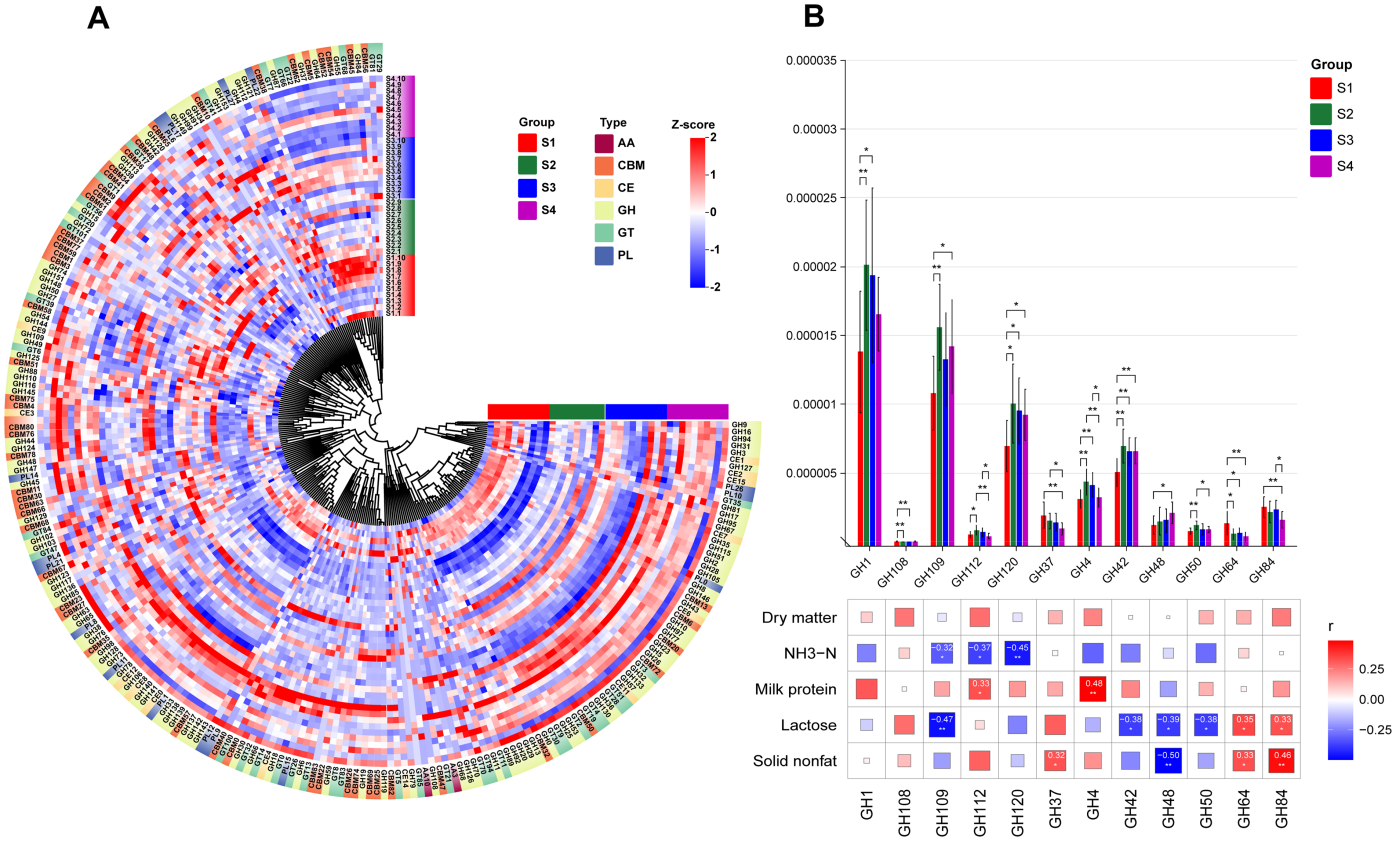
**(A)** HCA of CAZy enzyme classification identified in the rumen microbiota of S1–S4. The inner ring of color blocks represents rumen samples from cows of different groups, while the outer ring of color blocks corresponds to the six types of CAZy enzymes: AA, CBM, CE, GH, GT, and PL. **(B)** Spearman correlation analysis between differential GH enzymes identified in the rumen microbiota of S1–S4 and differential physiological indicators. * indicates *p* < 0.05, ** indicates *p* < 0.01, *** indicates *p* < 0.001.

The breakdown of complex carbohydrates by rumen microbiota affects rumen fermentation processes, relying on CAZymes. By comparing with the CAZymes database, we identified six enzyme types in both 1–4 parity Sanhe cows and Holstein cows, including auxiliary activities (AA), carbohydrate-binding modules (CBM), carbohydrate esterases (CE), glycoside hydrolases (GH), glycosyl transferases (GT), and polysaccharide lyases (PL). Among these, GH enzymes were the most abundant in both S and H groups (Supplementary Figure S1). Heatmap analysis of CAZymes abundance distribution in S revealed overall higher abundance in most CAZymes in S4 (Figure 5A). Comparing the inter-group differences in enzymes with higher abundance, we found significant differences in 12 GH enzymes. GH1, GH109, GH112, GH120, GH4, GH42, GH48, and GH50 showed higher abundance in multiparous cows (S2–S4), while GH108, GH37, GH64, and GH84 were more abundant in primiparous cows (S1). Correlation analysis using Spearman’s method between previously measured rumen and milk differential indicators and these differential GH enzymes revealed (Figure 5B): ammonia nitrogen showed significant negative correlations with GH109, GH112, and GH120. Milk protein exhibited significant positive correlations with GH112 and GH4. Lactose correlated with up to 6 GH enzymes, including significant positive correlations with GH64 and GH84, and significant negative correlations with GH109, GH42, GH48, and GH50. Fat-free dry matter exhibited significant negative correlations with GH48 and significant positive correlations with GH64 and GH84.

**Figure 6:**
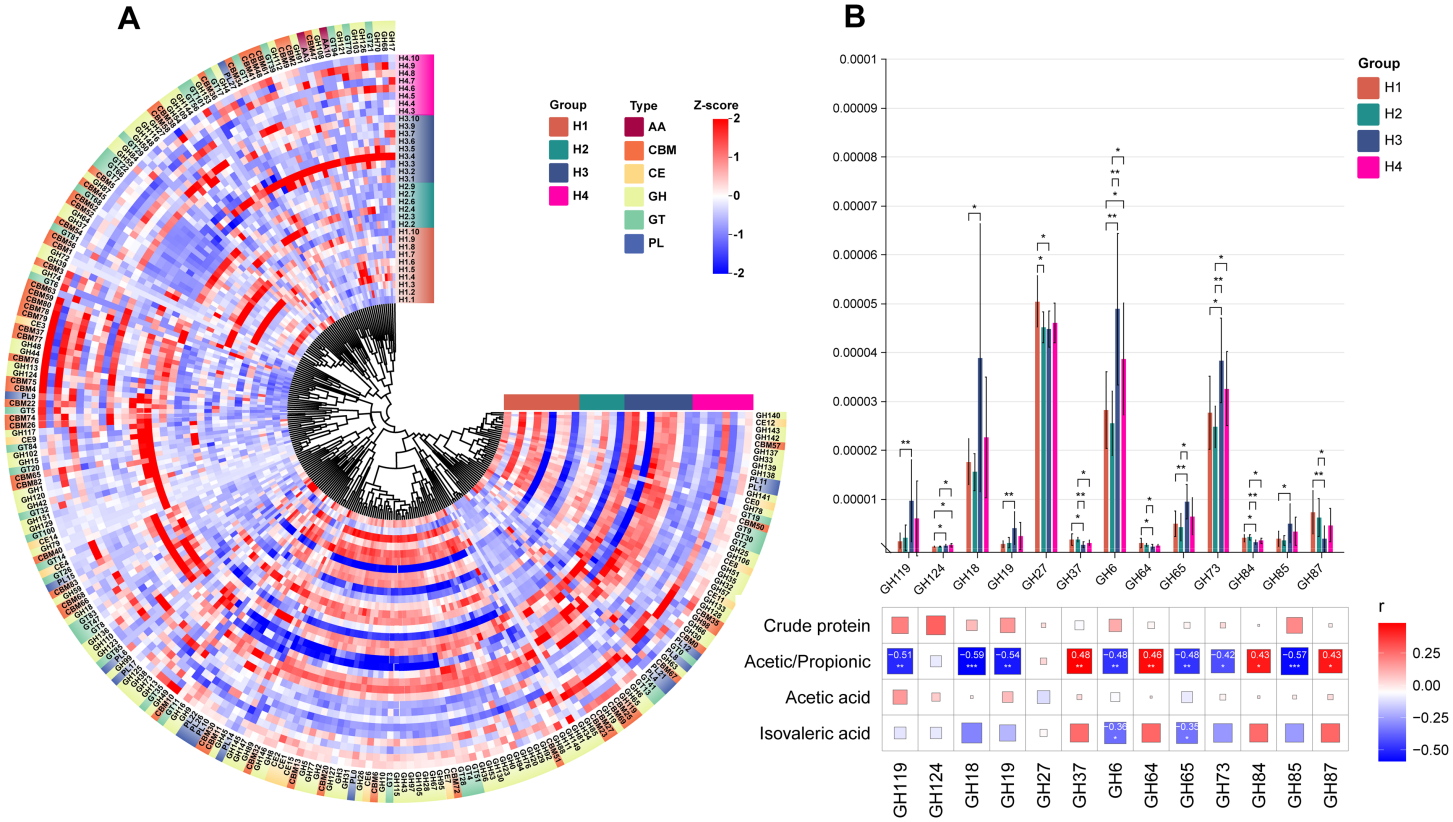
**(A)** HCA of CAZy enzyme classification identified in the rumen microbiota of H1–H4. The inner ring of color blocks represents rumen samples from cows of different groups, while the outer ring of color blocks corresponds to the six types of CAZy enzymes: AA, CBM, CE, GH, GT, and PL. **(B)** Spearman correlation analysis between differential GH enzymes identified in the rumen microbiota of S1–S4 and differential physiological indicators. * indicates *p* < 0.05, ** indicates *p* < 0.01, *** indicates *p* < 0.001.

For the H group, it can be observed that most CAZymes have higher abundance in H3 (Figure 6A). Additionally, we identified 13 GH enzymes (GH119, GH124, GH18, GH19, GH27, GH37, GH6, GH64, GH65, GH73, GH84, GH85, GH87) with significant inter-group differences (Figure 6B). Among these differential GH enzymes, H3 showed the highest abundance in GH119, GH18, GH6, GH173, and GH85, highlighting differences in enzyme abundance between H1/H2 and H3/H4. Correlation analysis with previously detected differential indicators revealed significant associations with these 13 GH enzymes: acetic/propionic acid showed significant positive correlations with GH37, GH64, GH84, and GH87, and significant negative correlations with the other 7 enzymes. Additionally, isovaleric acid exhibited significant negative correlations with GH6 and GH65, while lactose showed significant positive correlation with GH64.

## 4 Discussion

To fill the knowledge gap regarding the impact of rumen microecology on the production performance of Sanhe cattle, we focused on comparing the rumen microbiota of Sanhe cattle and Holstein cows across 1–4 parities. The aim was to identify potential differences in microbial composition and functional characteristics associated with parity and breed under the same dietary conditions, as well as their effects on lactation metabolism.

For Sanhe cattle, our previous studies found that the milk metabolome of S1–S4 displayed a pattern where S1 was significantly different from S2/S3/S4. This aligns with the observed changes in the rumen microecological environment in this study, where the microbial abundance and the types of enriched functional pathways in S1 also significantly differed from those in multiparous cows. We found that among the genera-level microbes significantly enriched in S1, both *Neocallimastix* and *Piromyces* are anaerobic fungi producing cellulase (17), mainly found in the rumen and intestines of ruminants (18, 19). *Neocallimastix* has a very high cellulase activity due to its 89% degradation activity on the cell wall (20). *Piromyces* can ferment cellulose to produce more acetate (21). Previous studies have found that using strains from these two genera for *in vitro* fermentation can increase DM digestibility and VFA content (22), which is consistent with the trends observed in S1 in this *in vivo* experiment. It is currently known that the genus *Prevotella* is the most abundant microbial group detected in ruminants globally (23). On one hand, *Prevotella* can contribute to rumen nitrogen metabolism by hydrolyzing proteins to produce ammonia (24, 25). On the other hand, as an ammonia-producing bacterium, it can generate ammonia through the deamination of amino acids (26), both of which promote the production of ammonia nitrogen. The concentration of NH_3_-N in the rumen reflects the relationship between the rate of ammonia nitrogen production and utilization in the rumen. Compared to *Prevotella ruminantium* and *Prevotella bryantii*, which have been verified to have a positive correlation with ammonia nitrogen production (27), the specific functions of the three *Prevotella*-related biomarkers identified in S1 are currently less studied. However, this study suggests that they may still be one of the reasons for the higher ammonia nitrogen levels in S1.

Although there were no significant differences in VFA levels between S1–S4, the total VFA and other short-chain fatty acids showed a higher trend in S1. Among the four biomarkers in S1, *Lactobacillus fructivorans* can decompose fructose to produce more acetic acid (28). The other three biomarkers are related to *Prevotella*, and previous studies have shown that the abundance of *Prevotella* is positively correlated with VFA production, improving fermentation efficiency and promoting the production of short-chain fatty acids (29–31). We also observed that the rumen microbiota in S1 was significantly enriched in KEGG pathways such as autophagy—animal, plant-pathogen interaction, and endocytosis, which are related to immune functions (32), cell metabolism (33), and intracellular material transport (34). These results suggest that the rumen microbiome in S1 undergoes dynamic adjustments to cope with the stress of the first lactation experience, showing more dramatic changes. In contrast, in S2/S3/S4, we observed a higher enrichment of ATP-binding cassette (ABC) transporters (K01990 and K02003) and ABC-2 type transport system permease protein (K01992) in the rumen microbiota, which aligns with the higher mapping of ABC transporters pathways in the KEGG database for S2/S3/S4. ABC transporters in microorganisms mainly participate in the transport of nutrients, especially monosaccharides and amino acids (35). These metabolic activities may indirectly affect the digestion, absorption, and nutrient metabolism of dairy cows, consistent with our previous findings of higher glucose and total protein levels in the serum of S2/S3/S4 cows.

Cows themselves cannot synthesize any enzymes necessary for the deconstruction of plant biomass. They rely primarily on the rumen microbiome to release energy from plant polysaccharides in the form of carbohydrates and sugars (36). Within the CAZyme family, glycoside hydrolases (GHs) are the most abundant and diverse group responsible for breaking glycosidic bonds in plant polysaccharides. They account for 50% of the classified enzymes in the CAZyme database (37) and can decompose cellulose, hemicellulose, and starch into simple carbohydrates that cows can absorb and utilize. Glucose and other substances produced by the breakdown of substrates such as cellulose by GH enzymes are important precursors for lactose synthesis (38, 39). Among the GH enzymes that showed a significant positive correlation with lactose content in Japanese Black cows, GH64 can hydrolyze β-1,3-D-glucan (40), releasing glucose monomers, a process providing the carbon source needed for lactose synthesis. GH84 can participate in the modification of glycoproteins (41), potentially regulating enzymes or substrates related to the lactose synthesis pathway and indirectly influencing lactose production. However, the actual abundance of these GH enzymes in the rumen, the downstream products they produce, and their impact on lactose synthesis through blood circulation still need to be verified through a series of experiments. This is necessary to further explore the mechanisms by which rumen microbial functions affect the biosynthetic pathways of milk components.

Compared to other breeds, Holstein cows are the most widely distributed high-yield dairy cattle globally, and research on their metagenome is both extensive and in-depth (42, 43). However, longitudinal comparisons of rumen microbiota changes with parity and cross-sectional comparisons of rumen microbiota under the same dietary conditions with other cattle breeds are still relatively scarce. Studies on Holsteins have shown that in multiparous cows, parity may be one of the driving factors for host-microbe interactions (44). Previous research mentioned that differences in microbial community composition are mainly attributed to diet, with the host having a smaller impact (23). This study indicates that under the same dietary conditions, the species composition structure of high-abundance phyla and genera in the rumen microbiota of Japanese Black cows and Holstein cows is similar, though the abundance varies slightly between different parities. We found that under the farm conditions we investigated, the trend of changes in the rumen microbiota of Holstein cows is somewhat different from that of Japanese Black cows, where S1 differs from S2/S3/S4. Specifically, H1 and H2 are similar, H3 and H4 are similar, but H1/H2 differ from H3/H4. Interestingly, these changes in the rumen microecology of Holstein cows align with the trends in metabolic profile changes we previously identified, showing similar patterns where H1 and H2 have closer abundance and differ from H3 and H4 on the heatmap (6). These findings suggest that rumen microbiota can influence body metabolism and thereby affect milk traits in different cattle breeds.

In terms of rumen fermentation, unlike S1, which had slightly higher levels of various VFAs than the other parities, the VFA changes in the H group did not follow a regular pattern. We found that although H3 had less acetate and more propionate in the rumen, the differences between groups were not significant. However, the acetate-to-propionate ratio in H3 was significantly lower than in the other parities. This could be related to the significantly higher abundance of *Succinivibrio* and *Ruminobacter* genera found in H3. Previous studies have shown that using a glucogenic diet as a substrate for *in vitro* fermentation can lead to a reduced acetate/propionate ratio, accompanied by higher abundances of *Succinivibrio* and *Ruminobacter* genera (45). Propionate can be produced directly via the decarboxylation of succinate, a pathway in which the *Succinivibrio* genus plays an important role (46). Moreover, we found that many GH enzymes in H3 had higher abundances and significant negative correlations with the acetate/propionate ratio. These GH enzymes do not directly participate in propionate production but can promote propionate production by providing monosaccharide substrates required for microbial fermentation. Among the GH enzymes highly expressed in H3, GH119 (47), GH6 (48), and GH65 (49) can hydrolyze α-glucan, cellulose, and oligosaccharides to produce glucose, which can promote propionate production (50). *s_Fibrobacter succinogenes* is a major degrader of lignocellulose substances in the intestines of herbivores (51). Previous studies have also found that lower levels of isovaleric acid accompanied by lower abundances of *s_Fibrobacter succinogenes* in the rumen of Altay sheep at different energy feeding levels (52). However, in Nellore calves, it was found that with an increase in concentrate content, rumen isovaleric acid concentration increased, but *s_Fibrobacter succinogenes* decreased (53). This is contrary to the higher isovaleric acid content accompanied by higher abundances of *s_Fibrobacter succinogenes* found in H1 in this study, which might be related to differences in feed composition and the stage of the cattle. *s_Eubacterium uniforme*, identified as a common biomarker in both H4 and S4, is a fiber-digesting bacterium that mainly decomposes cellobiose and xylan and has previously only been isolated from sheep rumen (54).

## 5 Conclusion

This study explored the differences in rumen microbiota composition between multiparous Sanhe cattle and Holstein cows and found two main points: First, under the same dietary conditions, the species composition of the rumen microbiota was similar between Sanhe cattle and Holstein cows, but their abundances differed. Second, the rumen microecological patterns were highly correlated with milk metabolic patterns, but breed remained a decisive factor influencing dairy cow performance. These results highlight the breed-specific metabolic adaptability of dairy cows, which changes with parity, emphasizing the dynamic interactions between genetic background, physiological state, and metabolic regulation. Understanding these complex metabolic dynamics is crucial for optimizing feeding strategies, improving production efficiency, and ensuring the overall health and welfare of dairy herds.

## Data Availability

The datasets presented in this study can be found in online repositories. The names of the repository/repositories and accession number(s) can be found in the article/Supplementary material.

## Footnotes

^1^ http://jgi.doe.gov/data-and-tools/bbtools/

^2^ https://www.omicstudio.cn/tool

^3^ http://www.cazy.org/

^4^ http://csbl.bmb.uga.edu/dbCAN/

^5^ https://www.chiplot.online/index.html
