# Supplementary material for: The impact of rumen microbial composition on apparent digestibility, rumen fermentation and metabolism in Sanhe cows and Holstein cows of different parities under identical dietary conditions
Source: Front Vet Sci. 2025 Feb 17;11:1463209. doi: 10.3389/fvets.2024.1463209 (PMC11873279; doi:10.3389/fvets.2024.1463209)
Supplement: Supplementary file 1 [file Table_1.docx]

Supplementary Material

## Supplementary Figures


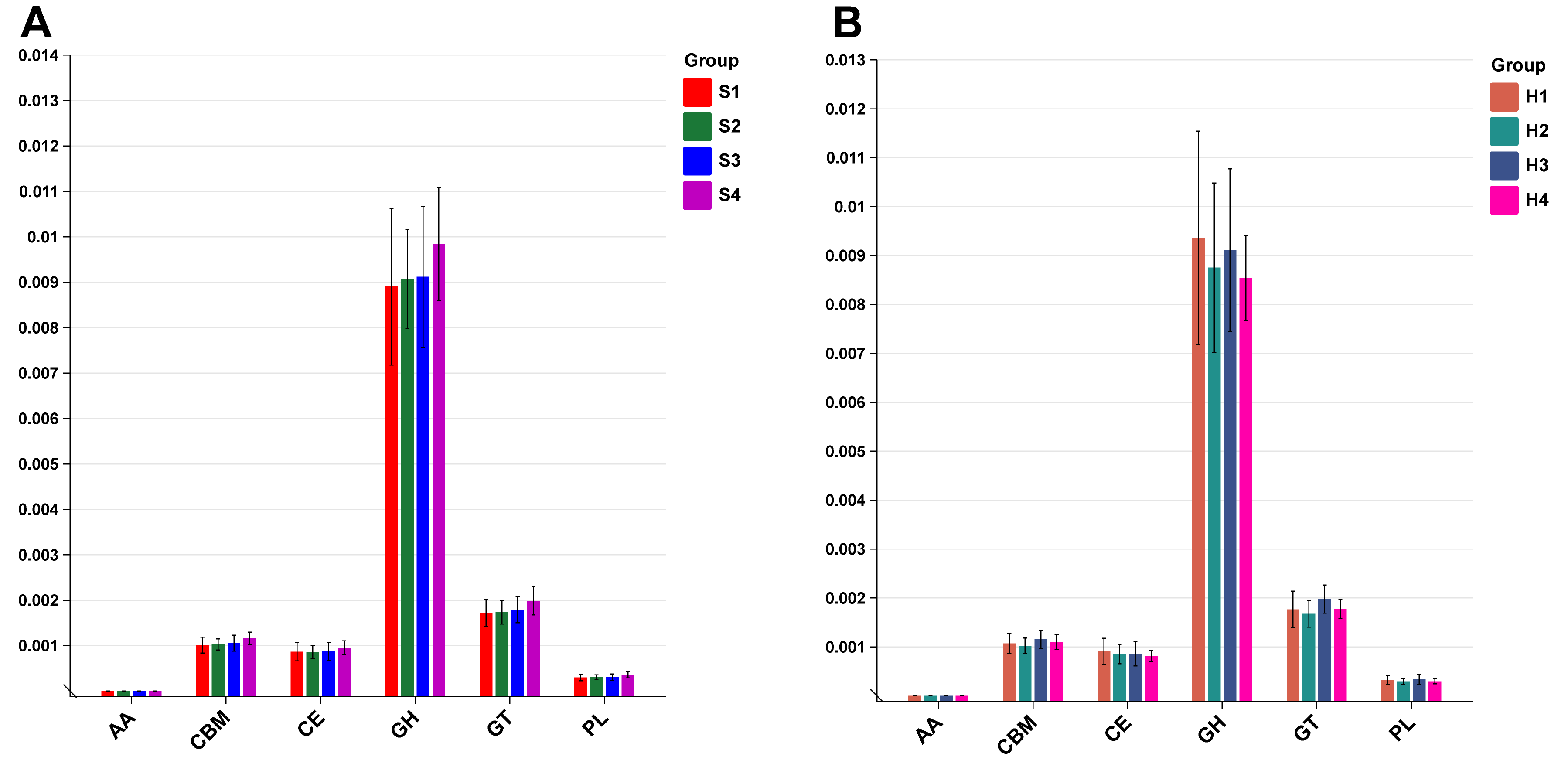


**Supplementary Figure 1.** (A) Types and abundance of CAZy enzymes identified in the rumen microbiota of S1-S4. (B) Types and abundance of CAZy enzymes identified in the rumen microbiota of H1-H4.
